# Unexpected Fine-Scale Population Structure in a Broadcast-Spawning Antarctic Marine Mollusc

**DOI:** 10.1371/journal.pone.0032415

**Published:** 2012-03-05

**Authors:** Joseph I. Hoffman, Andy Clarke, Melody S. Clark, Peter Fretwell, Lloyd S. Peck

**Affiliations:** 1 Department of Animal Behaviour, University of Bielefeld, Bielefeld, Germany; 2 British Antarctic Survey, Natural Environment Research Council, Cambridge, United Kingdom; Biodiversity Insitute of Ontario - University of Guelph, Canada

## Abstract

Several recent empirical studies have challenged the prevailing dogma that broadcast-spawning species exhibit little or no population genetic structure by documenting genetic discontinuities associated with large-scale oceanographic features. However, relatively few studies have explored patterns of genetic differentiation over fine spatial scales. Consequently, we used a hierarchical sampling design to investigate the basis of a weak but significant genetic difference previously reported between Antarctic limpets (*Nacella concinna*) sampled from Adelaide and Galindez Islands near the base of the Antarctic Peninsula. Three sites within Ryder Bay, Adelaide Island (Rothera Point, Leonie and Anchorage Islands) were each sub-sampled three times, yielding a total of 405 samples that were genotyped at 155 informative Amplified Fragment Length Polymorphisms (AFLPs). Contrary to our initial expectations, limpets from Anchorage Island were found to be subtly, but significantly distinct from those sampled from the other sites. This suggests that local processes may play an important role in generating fine-scale population structure even in species with excellent dispersal capabilities, and highlights the importance of sampling at multiple spatial scales in population genetic surveys.

## Introduction

A classical paradigm in marine population biology is that broadcast-spawning species exhibit little or no genetic structure relative to otherwise ecologically equivalent brooders [1]. However, despite this notion having received substantial empirical support [2], [3], [4], [5], [6], [7] a number of exceptions have also been documented. For example, several species of brooding corals [8] and amphipods [9] have been found to be genetically unstructured over large areas, highlighting the potential for intermittent long-distance dispersal, perhaps mediated by violent storms. This suggests the need for more studies aimed at gaining a broader understanding of the conditions under which marine species deviate from theoretical expectations.

An interesting case in point is provided by the Antarctic limpet, *Nacella concinna*, one of the most abundant and widespread of all shallow-water Antarctic marine macro-invertebrates [10]. This species has long been regarded as a classic example of a broadcast-spawner, possessing free-swimming planktotrophic veliger larvae that can survive in the water column for up to two months [11]. However, contrary to expectations, several lines of evidence point towards populations of this species being spatially structured. For example, de Arazamendi et al. [12] reported statistically significant genetic differences between intertidal and subtidal morphs of this species within a single locality using 35 binary inter-simple sequence repeat (ISSR) markers, although two subsequent studies using larger panels of Amplified Fragment Length Polymorphism (AFLP) loci were unable to replicate this finding at other locations [13], [14]. Similarly, Beaumont and Wei [15] detected genetic differences between limpets from the South Orkney Islands and South Georgia using five allozymes, while more recently Hoffman et al. [16] found surprisingly strong population structure using AFLPs along a latitudinal gradient spanning the Antarctic Peninsula and the outlying islands of Signy and South Georgia. In the latter study, the strongest genetic differences were observed between islands separated by deep ocean currents, whereas all but one of the Peninsula sites were genetically indistinguishable from one another. The exception was Adelaide Island, situated near the base of the Antarctic Peninsula, which was found to be weakly differentiated (*F*_st_ = 0.003–0.007, *P*<0.05) from the other Peninsula sites.

The genetic distinctness of Adelaide Island poses a conundrum due to the absence of any obvious oceanographic barriers to gene flow in this region. One possible explanation could be that larval transport between Galindez and Adelaide Islands is restricted by some form of previously overlooked, large-scale hydrological barrier such as a gyre or eddy system. This is plausible given that the Antarctic Circumpolar Current runs northwards along the Peninsula, the Antarctic Coastal Current runs southwards closer to the shore and there are indications of a series of semi-isolated gyres between the two [17]. Another possibility is that current systems within Ryder Bay at the base of the Antarctic Peninsula could be sufficiently strong to impart mild genetic structure by retaining larvae within localized areas. Finally, highly heterogenous glacier coverage within Ryder Bay appears to have generated a patchwork of habitats of greatly varying age, with some locations around Rothera Point being as young as 100 years old whereas others like Anchorage Island may be thousands of years old [18]. Consequently, it is possible that populations across the region could have experienced markedly different larval inputs as well as demographic histories. Of particular importance could be founder effects and population bottlenecks, which can profoundly influence rates of genetic drift.

**Figure 1:**
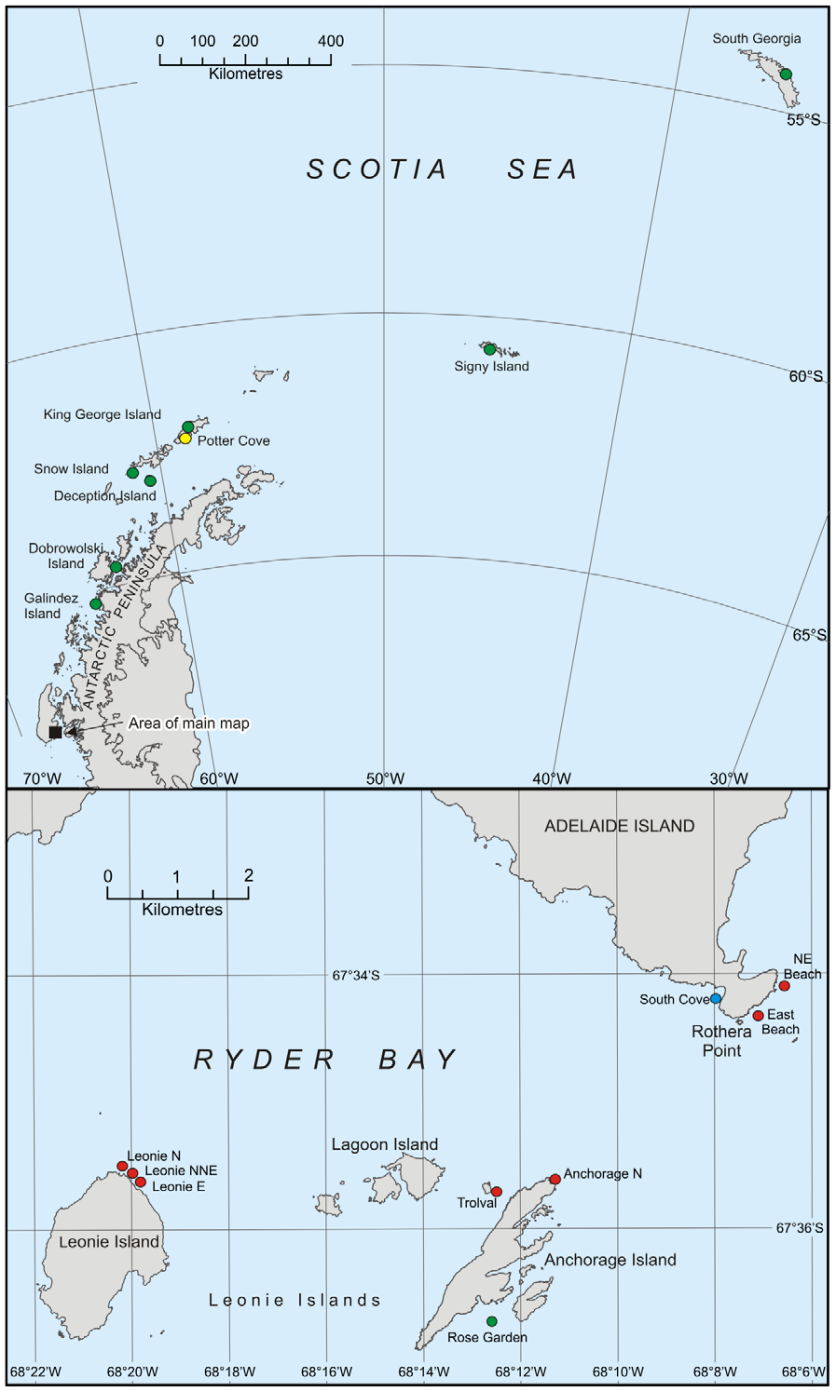
Map showing *N. concinna* sampling locations, including populations sampled by three previous studies [**12]**, [13], [16****]. The upper panel shows the Antarctic Peninsula with sites studied by Hoffman et al. [16] and de Arazamendi et al. [12] highlighted in green and yellow respectively. The lower panel shows the Ryder Bay area (Adelaide Island) with the green point denoting Rose garden, sampled by Hoffman et al. [16], and the blue point representing another previous study at Rothera [13]. Locations sampled and analysed as part of the current study are denoted by red points.

To further explore the population genetic structure of *N.concinna*, we designed a hierarchical sampling strategy embracing three separate regions within Ryder Bay, each sub-sampled three times to facilitate the detection of any potential fine-scale differences (Figure 1). We then used Amplified Fragment Length Polymorphisms (AFLPs) to generate large numbers of highly reproducible binary markers capable of resolving even relatively minor genetic differences [19], [20], [21], [22]. Previously published AFLP data from the closest of the Antarctic Peninsula populations, Galindez Island, were included for comparison [16]. We made the following simplistic predictions: (i) if genetic exchange between Adelaide and Galindez Islands is restricted by a large-scale gyre or eddy system, we would expect the Ryder Bay populations to differ from Galindez but to be themselves genetically homogenous; (ii) if local currents play an important role in mediating larval transport and deposition, we would expect to observe genetic differences among the populations within Ryder Bay, with some or all of these populations also differing from Galindez; (iii) if habitat age is the driving factor, the greatest genetic differences should involve sites differing maximally in age.

## Materials and Methods

### Tissue sample collection and DNA extraction

Antarctic limpets were collected by SCUBA divers during the austral summer of 1999 from the shallow sublittoral zone. A hierarchical sampling strategy was employed embracing nine sites sampled from Ryder Bay, Adelaide Island (see Table 1 and Figure 1 for sampling locations). Previously published AFLP data from Galindez Island [16] were also included for comparison. Tissue samples were stored in 95% ethanol, initially for four months at −20°C and thereafter at room temperature. For each specimen, total genomic DNA was extracted from a small piece of foot tissue using the Qiagen DNeasy tissue extraction kit following the manufacturer's recommended protocols. Unfortunately, high-quality DNA could not be obtained from any of the individuals collected from Leonie North, probably due to these samples having inadvertently dried out during storage. Although we still subjected these individuals to the AFLP procedure, they did not yield interpretable PCR products and were therefore excluded from subsequent analyses.

**Table 1:** Details of sampling locations and numbers of *N. concinna* individuals collected for genetic analysis at each site.

Region	Site	Population	Latitude (S)	Longitude (W)	Depth (m)	No. of samples collected	No. of samples successfully genotyped
Adelaide Island	Rothera Point	South Cove	−67.5700	−68.1320	25	48	48
		East Beach	−67.5720	−68.1180	28	46	46
		North East Beach	−67.5667	−68.1000	21	45	45
	Anchorage Island	Trolval	−67.6081	−68.2181	10	37	37
		Anchorage North	−67.6017	−68.2017	25	46	46
		Rose Garden	−67.6069	−68.1911	20	48	48
	Leonie Island	Leonie East	−67.6047	−68.3292	18	45	45
		Leonie North North East	−67.6025	−68.3358	25	42	42
		Leonie North	−67.5978	−68.3378	25	48	0
Galindez Island	–	–	−65.2333	−64.2333	14	48	48
	Total					453	405

### AFLP genotyping and estimation of the genotyping error rate

Our AFLP protocol was adapted from Vos et al. [23] and is described in detail by Dasmahapatra et al. [24]. Seven different selective primer combinations were employed (Table 2). PCR products were resolved by electrophoresis on standard 6% polyacrylamide sequencing gels and detected by autoradiography. Exposed X-ray films were assessed and if required, a second exposure was made for an adjusted time period. Gels were scored by eye and genotypes were entered manually into a Microsoft excel spreadsheet. The genotyping error rate was estimated for the resulting dataset following Hoffman and Amos [25] by independently re-extracting, re-genotyping and blind-scoring 28 individuals (approximately 7% of the samples). The error rate per reaction was quantified as the number of mismatching genotypes divided by the number of polymorphic bands compared [26].

**Table 2:** Primer combinations used for the AFLP selective amplification and numbers of AFLP loci generated.

*Taq*I primer (5′-3′)	*Eco*RI primer (5′-3′)	Total number loci	Number of polymorphic loci
GATGAGTCCTGACCGA–CTG	GACTGCGTACCAATTC–AGC	33	26
GATGAGTCCTGACCGA–CGA	GACTGCGTACCAATTC–AGC	29	20
GATGAGTCCTGACCGA–CAG	GACTGCGTACCAATTC–AGC	8	5
GATGAGTCCTGACCGA–CAC	GACTGCGTACCAATTC–AGC	30	25
GATGAGTCCTGACCGA–CAC	GACTGCGTACCAATTC–ATG	43	37
GATGAGTCCTGACCGA–CCA	GACTGCGTACCAATTC–AAC	34	27
GATGAGTCCTGACCGA–CCA	GACTGCGTACCAATTC–ACA	17	15
Total		194	155

### Data analyses

The *N. concinna* AFLP dataset consisted of 62, 775 binary characters representing the presence and absence genotypes of 405 individuals at 155 polymorphic AFLP loci. To explore patterns of genetic differentiation, we first calculated pairwise *F*_st_ values and associated *P*-values among all of the sites using the program Aflp-Surv [27]. To compensate for the large number of statistical tests being carried out, the resulting *P*-values were corrected using the False Discovery Rate (FDR) approach of Benjamini and Hochberg [28]. Aflp-Surv was also used to conduct an overall test of genetic differentiation using 10,000 permutations of the dataset. To relate genetic differences among the sites to their geographic proximity, we next carried out an isolation-by-distance analysis using geographic distances calculated within a Geographic Information System (ESRI ArcGis v 9.2). By applying distance allocation tools to a bathymetric digital elevation model (GEBCO: General Bathymetric Chart of the Ocean 2003) and excluding land from the analysis, the shortest sea-route between each site was calculated. A Lambert Conformal Conic projection was used to ensure the least possible spatial distortion. The significance of correlations between genetic and geographic distance matrices was assessed using Mantel tests with 999 iterations implemented in Genalex v6 [29].

## Results

To explore the fine-scale hierarchical population genetic structure of *N.concinna* within Ryder Bay, Adelaide Island, a total of 405 individuals from nine sites were genotyped at seven selective AFLP primer combinations (Table 1). This yielded 194 loci that could be scored unambiguously in all of the individuals, of which 155 (79.9%) were polymorphic (Table 2). The genotyping error rate was estimated to be low at 0.011 (45 differences were observed out of 4285 comparisons). Of the discrepancies observed between the two sets of genotypes, 18 (40.0%) and 1 (2.2%) were attributed to scoring and data entry errors respectively, while the remaining 26 (57.8%) were due to the stochastic appearance or disappearance of bands. The latter has been previously documented at a similar level by Bonin *et al.* [26].

Population genetic differentiation was weak but statistically significant overall (*F*_st_ = 0.0004, *P* = 0.011). *F*_st_ values obtained in pairwise comparisons among the sites were also low (Table 3), although seven values were individually significant (six at *P*<0.01 and one at *P*<0.05) and a further seven approached significance (0.08<*P*<0.05). Following table-wide FDR correction for multiple tests [28], six *F*_st_ values remained significant at *P*<0.05. No relationship was observed between the shortest geographic distance by sea and genetic distance (Mantel's r = −0.181, *n* = 9, *P* = 0.347). However, significant *P*-values were only encountered among pairwise comparisons involving sites from Anchorage Island, an imbalance that is itself statistically significant (7/21 versus 0/15, Binomial proportions test, *P* = 0.039).

**Table 3:** Pairwise *F*_st_ values among *N. concinna* sampled from nine different sites (above diagonal).

	South Cove	East Beach	North East Beach	Trolval	Anchorage North	Rose Garden	Leonie East	Leonie North North East	Galindez Island
South Cove	*	0.001	0.000	0.004	0.002	0.003	0.000	0.000	0.001
East Beach	0.123	*	0.000	0.000	0.000	0.005	0.000	0.000	0.000
Northeast Beach	0.265	0.522	*	0.002	0.000	0.003	0.000	0.000	0.000
Trolval	***0.013***	0.523	0.078	*	0.002	0.006	0.005	0.000	0.003
Anchorage North	0.058	0.324	0.853	0.054	*	0.005	0.001	0.000	0.000
Rose Garden	0.054	***0.009***	0.058	***0.005***	***0.005***	*	0.008	0.003	0.003
Leonie East	0.244	0.214	0.523	***0.008***	0.109	***0.001***	*	0.000	0.000
Leonie North North East	0.442	0.900	0.913	0.306	0.824	**0.038**	0.270	*	0.000
Galindez Island	0.143	0.803	0.820	0.052	0.712	0.059	0.514	0.490	*

*P*-values, calculated using 10,000 permutations of the dataset, are given below the diagonal.

Significant values without correction for multiple statistical tests (*P*<0.05) are highlighted in bold and values closely approaching significance (0.08<*P*<0.05) are underlined. *P*-values that remained significant after controlling for the false discovery rate are italicised (see Materials and methods for details).

## Discussion

In this study, we combined a highly informative panel of AFLPs with a hierarchical sampling design within Ryder Bay to explore the fine-scale population structure of Antarctic limpets (*Nacella concinna*) near the base of the Antarctic Peninsula. Limpets from Anchorage Island were found to be subtly, but significantly distinct from those sampled from the other sites, with implications for understanding the population genetic connectivity of marine species and more generally for the design of population genetic surveys.

### Pattern and magnitude of genetic differentiation

Antarctic limpets are prolific broadcast-spawners with relatively long-lived planktotrophic veliger larvae and high larval densities [11], [30], [31]. Consequently, *a priori* we favoured a large-scale mechanism to explain the previously reported genetic difference between limpets from Adelaide and Galindez Islands [16]. It was therefore surprising to find genetic differences, however weak, between sites within Ryder Bay. It is important to stress that these differences are very small (pairwise *F*_st_ values never exceeded 0.008) and hence that statistical significance may only have been reached due to a combination of large sample sizes of individuals (average *n* = 45) and polymorphic loci (*n* = 155). The demographic consequences of such subtle genetic differences can be difficult to assess [32], partly due to the problem of sampling error associated with the measurement of *F*_st_, although this should decrease with increasing numbers of markers. Nevertheless, there is good reason to believe that the differences we report are genuine. First, efforts were made to drive the genotyping error rate downwards by scoring only bands that could be clearly distinguished as either present or absent in all individuals, resulting in an overall error rate of only 0.011 per reaction. Second, not only was overall *F*_st_ significant in a global permutation test, but also all but one of the significant values obtained in pairwise population comparisons were robust to table-wide FDR correction. Third, if most or all of the significant tests obtained were attributable to Type I error, they should be distributed randomly, which was clearly not the case in this study. Individually significant *F*_st_ values were only found in pairwise comparisons involving Anchorage Island (Binomial proportions test, *P*<0.05) and this pattern became even stronger when marginally significant *P*-values (0.08<*P*<0.05) were taken into account (14/21 versus 0/15 for comparisons excluding Anchorage; Binomial proportions test, *P* = 0.0002).

It is also helpful to consider our results in the context of a previous study spanning most of the Antarctic Peninsula and the islands of Signy and South Georgia [16]. Using the same AFLP marker panel and similar sample sizes, Signy and South Georgia were found to be moderately differentiated from the Antarctic Peninsula sites (mean *F*_st_ = 0.028 and 0.114 respectively), consistent with their being separated by deep ocean channels that may act as a barrier to larval transport. In contrast, five sites sampled from Antarctic Peninsula (Dobrowolski, Deception, Snow and King George Islands) were genetically indistinguishable from one another, yielding *F*_st_ values of zero in all ten pairwise comparisons. This further reinforces the assertion that significant positive values obtained in comparisons involving Anchorage Island are genuine.

A previous study by de Aranzamendi et al. [12] similarly reported fine-scale genetic differences between *N. concinna* populations at Potter Cove, King George Island, on the South Shetland Islands, although these primarily involved comparisons between the intertidal and subtidal morphs, which differ markedly from one another in shell size and physiology [33], [34]. Based on 35 inter-simple sequence repeat (ISSR) markers, these authors reported *F*_st_ values almost an order of magnitude higher than those found in this study, despite sample sizes being far smaller (total *n* = 108 individuals distributed over 7 populations). However, two subsequent studies conducted independently at other localities found no genetic differences between intertidal and subtidal *N. concinna* individuals using larger panels of AFLPs [13], [14]. Furthermore the two morphs have also recently been shown to be part of a continuous cline in both morphology and physiology with depth [13], [35]. Nevertheless, we took the precaution of ensuring that depth could not be a confounding factor in the current analysis by focusing exclusively on subtidal individuals.

### Possible explanations for fine-scale population structuring

Although relatively few studies have explored the potential for fine-scale genetic structure in Antarctic marine organisms, the limited evidence available suggests that straits of deep water as narrow as 30km can significantly impact population connectivity [16], [36]. For brooders, deep channels probably provide an effective barrier to adult migration, whereas in broadcast-spawners a more likely explanation is that fast-flowing currents carry away dispersing larvae. However, deep water cannot explain the results of this study because Ryder Bay is uniformly shallow, attaining a maximum depth of only around 500 metres [37]. Instead, we considered three primary explanations, which are detailed at the end of the introduction. The first of these, interrupted gene flow between Adelaide and Galindez Islands, is not supported by our data given that Anchorage Island appears to comprise a genetically distinct ‘pocket’ within the Ryder Bay region, with limpets sampled from all of the other sites including Galindez Island being indistinguishable from one another. This pattern could easily have been overlooked by the previous large-scale study if, for example, we had originally sampled from Rothera and not Rose Garden. Consequently, we advocate the use of hierarchical sampling designs in population genetic surveys because, although greater sampling and experimental effort is required, the possibility of fine-scale structure can be accounted for.

The second possible mechanism based on local currents is more difficult to evaluate, but could potentially involve different types of water flow. The first of these, tidal currents, move particles relatively short distances, in the order of hundreds of metres along the shore and back again. In the abalone *Haliotis rubra*, tidal currents may be sufficient to impart fine-scale population structure due to an unusual behavioural pattern in which the larvae synchronise swimming with periods of low or no water flow [38]. However, no such behaviours have been identified in *N. concinna* and, unless tidal flows differ markedly across Ryder Bay, this would in any case be expected to generate uniform fine-scale structuring. A second possibility is that coastal eddies around Anchorage Island could be advecting larvae back towards the shore, a mechanism invoked to explain local ‘hot spots’ of larval retention encountered in computer simulations [39]. Alternatively, external currents that deliver larvae into Ryder Bay could be important. Flow along this region of the Antarctic Peninsula is predominantly from north to south [40], and would therefore be expected to deliver larvae from localities along the northern Antarctic Peninsula. However, relatively shallow currents have also been documented that flow during winter from the Fallières Coast on the Antarctic Peninsula northwards towards Ryder Bay [40]. These could bring larvae from sites to the South of Ryder Bay that were not surveyed by Hoffman et al. [16] and which could potentially differ genetically. Because *N. concinna* spawns in the early austral summer, with larvae present in the water column throughout late summer and early winter [41], [42], the period of northward flow may not overlap with the pelagic phase of this species in many or even most years. However, winter currents from the south can be highly variable and the sites we analysed from Anchorage Island are situated on the outermost edge of Ryder Bay. Consequently, sporadic larval input to these exposed locations could potentially contribute to the genetic differences observed, especially if this occurred over lengthy timescales (see paragraph below). From this, it would be expected that further surveys to the south of Adelaide Island would find limpet populations that are genetically distinct from those to the north.

A third possibility relates to the fact that permanent ice cover and ice scour can profoundly impact communities of relatively sessile benthic Antarctic organisms. Coastal glaciers and ice shelves have been retreating for some time along the Antarctic Peninsula [43] with dramatic consequences for nearshore ecosystems [44]. Within Ryder Bay, detailed lichenographic studies suggest that the retreat of ice has been uneven, leaving behind a patchwork of habitats of varying ages. This could have led to some sites having experienced different larval inputs to others, particularly if the main currents delivering larvae to the area vary over time. Spatial variation in the ebb and flow of sea ice might also directly impact the demography of local populations by enforcing sequential bottlenecks, which can accelerate genetic drift. In this regard, we find it intriguing that the genetically distinct Anchorage Island populations originate from the oldest site, which may be thousands of years older than habitats present around Rothera Point [18]. However, with so few populations studied, any potential link between habitat age and population structure should at this stage be viewed as speculative.

Finally, it is important to note that temporal stochasticity has also been implicated as a factor that could drive fine-scale population structure in planktonically dispersing marine invertebrates [39], [45]. This could reflect either spatiotemporal variation in selection on larvae [46] or extreme variability in the reproductive success of different individuals arising from the chance matching of spawning with suitable oceanographic conditions for fertilization, larval transport, deposition and recruitment [45]. However, to evaluate this possibility would require the incorporation of a temporal element into our sampling design. This hypothesis also predicts that specific cohorts of larvae or recruits should each represent only small subsets of the total genetic variation present within the population [45]. It would therefore be interesting to conduct large-scale genetic screening of larval cohorts [45], although both the experimental effort involved and the technological hurdles to be overcome would likely be considerable.

### Conclusions

Using a large panel of AFLP markers, we detected weak fine-scale population genetic structure among Antarctic limpets sampled from Ryder Bay. Although the underlying mechanisms as yet remain unclear, our study emphasises the importance of sampling over multiple spatial scales. It also contributes to a growing list of factors including differential microhabitat use [47], larval or adult behaviour [38] and fine-scale spatiotemporal variability in the physical environment [48] that may influence the population structure of marine invertebrates. Exploring the role of these and other factors should provide a fertile area for future research.
